# Impact of chronic liver disease upon admission on COVID-19 in-hospital mortality: Findings from COVOCA study

**DOI:** 10.1371/journal.pone.0243700

**Published:** 2020-12-10

**Authors:** Raffaele Galiero, Pia Clara Pafundi, Vittorio Simeon, Luca Rinaldi, Alessandro Perrella, Erica Vetrano, Alfredo Caturano, Maria Alfano, Domenico Beccia, Riccardo Nevola, Raffaele Marfella, Celestino Sardu, Carmine Coppola, Ferdinando Scarano, Paolo Maggi, Pellegrino De Lucia Sposito, Laura Vocciante, Carolina Rescigno, Costanza Sbreglia, Fiorentino Fraganza, Roberto Parrella, Annamaria Romano, Giosuele Calabria, Benedetto Polverino, Antonio Pagano, Carolina Bologna, Maria Amitrano, Vincenzo Esposito, Nicola Coppola, Nicola Maturo, Luigi Elio Adinolfi, Paolo Chiodini, Ferdinando Carlo Sasso, on behalf of COVOCA Study Group

**Affiliations:** 1 Department of Advanced Medical and Surgical Sciences, University of Campania “Luigi Vanvitelli”, Naples, Italy; 2 Medical Statistics Unit, Department of Physical and Mental Health and Preventive Medicine, University of Campania “Luigi Vanvitelli”, Naples, Italy; 3 Task Force Covid-19 Regione Campania, Napoli, Italy; 4 Internal Medicine, Sant’Ottone Frangipane Hospital, Ariano Irpino, Italy; 5 COVID Center "S. Anna e SS. Madonna della Neve" Hospital, Boscotrecase, Italy; 6 U.O.C. Infectious and Tropical Diseases, S. Anna e S. Sebastiano Hospital, Caserta, Italy; 7 Covid Center—Maddaloni Hospital, Maddaloni, Italy; 8 General Medicine Unit, Loreto Mare Hospital, Naples, Italy; 9 U.O.C. Infectious Diseases and Neurology, Cotugno Hospital, Naples, Italy; 10 U.O.C. Infectious Diseases of the Elderly, Cotugno Hospital, Naples, Italy; 11 U.O.C. Anestesia and Intensive Care Unit, Cotugno Hospital, Naples, Italy; 12 U.O.C. Respiratory Infectious Diseases, Cotugno Hospital, Naples, Italy; 13 U.O.C. Pneumology, Moscati Hospital, Avellino, Italy; 14 IX^th^ Division of Infectious Diseases and Interventional Ultrasound, Cotugno Hospital, Naples, Italy; 15 "Giovanni da Procida" Hospital, Salerno, Italy; 16 Emergency and Acceptance Unit, "Santa Maria delle Grazie" Hospital, Pozzuoli, Italy; 17 Internal Medicine Unit, Ospedale Del Mare, Naples, Italy; 18 U.O.C. Internal Medicine—Moscati Hospital, Avellino, Italy; 19 IV^th^ Division of Immunodeficiency and Gender Infectious Diseases, Cotugno Hospital, Naples, Italy; 20 Department of Mental Health and Public Medicine, Centro COVID A.O.U. Vanvitelli, Naples, Italy; 21 U.O.S.D. Infectious Diseases Emergency and Acceptance, Cotugno Hospital, Naples, Italy; National Institute for Infectious Diseases Lazzaro Spallanzani-IRCCS, ITALY

## Abstract

**Background:**

Italy has been the first Western country to be heavily affected by the spread of SARS-COV-2 infection and among the pioneers of the clinical management of pandemic. To improve the outcome, identification of patients at the highest risk seems mandatory.

**Objectives:**

Aim of this study is to identify comorbidities and clinical conditions upon admission associated with in-hospital mortality in several COVID Centers in Campania Region (Italy).

**Methods:**

COVOCA is a multicentre retrospective observational cohort study, which involved 18 COVID Centers throughout Campania Region, Italy. Data were collected from patients who completed their hospitalization between March-June 2020. The endpoint was in-hospital mortality, assessed either from data at discharge or death certificate, whilst all exposure variables were collected at hospital admission.

**Results:**

Among 618 COVID-19 hospitalized patients included in the study, 143 in-hospital mortality events were recorded, with a cumulative incidence of about 23%. At multivariable logistic analysis, male sex (OR 2.63, 95%CI 1.42–4.90; p = 0.001), Chronic Liver Disease (OR 5.88, 95%CI 2.39–14.46; p<0.001) and malignancies (OR 2.62, 95%CI 1.21–5.68; p = 0.015) disclosed an independent association with a poor prognosis, Glasgow Coma Scale (GCS) and Respiratory Severity Scale allowed to identify at higher mortality risk. Sensitivity analysis further enhanced these findings.

**Conclusion:**

Mortality of patients hospitalized for COVID-19 appears strongly affected by both clinical conditions on admission and comorbidities. Originally, we observed a very poor outcome in subjects with a chronic liver disease, alongside with an increase of hepatic damage.

## Introduction

After SARS-CoV-2 disease (COVID-19) outbreak in Wuhan in December 2019, Italy has been the first Western country heavily affected by the spread of the infection. Our country was among the pioneers of the clinical management of pandemic for all other European and non-European National Healthcare Systems (NHS), as undergone to a stress test for both healthcare and economic systems [1].

World reports stated that approximatively 20–51% of patients had at least one pre-existing disease on admission, among which the most prevalent were diabetes (10–20%), hypertension (10–15%), cardiovascular and cerebrovascular diseases (7–40%), liver disease (2–11%) [2,3]. The relationship between pre-existent comorbidities and adverse clinical outcome has been largely investigated. However, data are still partially controversial [2,4–9]. Particularly, several papers have reported a positive association between preexisting chronic liver diseases and either the risk of infection or the hospitalization of COVID-19 patients. The evidence of an association between liver disease and mortality among hospitalized patients is still poor [2,10].

To date, while waiting for a vaccine and specific therapies, and therefore to define the best clinical management, the identification of patients at the highest risk seems mandatory.

Aim of this study is to identify comorbidities associated with in-hospital mortality in several COVID Centers in Campania Region (Italy), with a particular focus on chronic liver disease.

## Materials and methods

### Study design and participants

COVOCA (observational study on the **COV**ID-19 population h**O**spitalized in **CA**mpania Region) is a retrospective observational cohort study, which involved 18 COVID centers throughout Hospitals of Campania Region, Italy. In depth, 11 were sub-intensive COVID-19 Units, 6 low intensive adapted with respiratory devices and 1 Intensive Care Unit.

**Fig 1:**
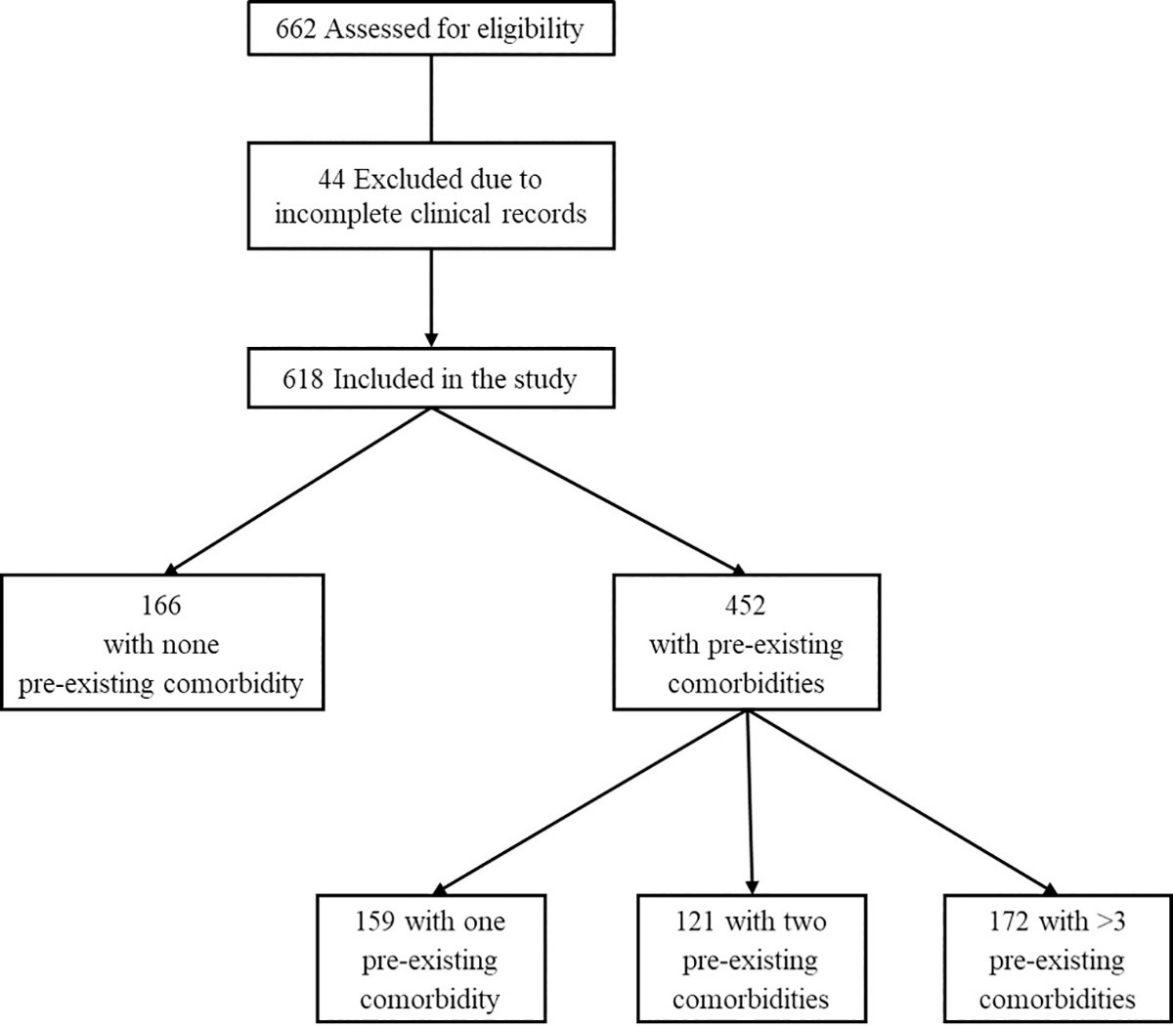
Flow-chart of the study.

We included all adult patients (≥ 18 years) with laboratory confirmed SARS-CoV-2 infection, who completed their hospitalization (discharged or dead) in the period between March 13, 2020 and July, 6 2020, of whom clinical records were available. All patients with either incomplete or missing clinical and laboratory data at baseline were excluded from the study. Flow-chart is shown in Fig 1. Data were accessed from July 10, 2020.

Clinical charts and hospital electronic records were used as data sources. All data were fully anonymized by the participating centers before being accessed. The study was approved by the local Ethics Committee (University of Campania Luigi Vanvitelli) and is in accordance with 1976 Declaration of Helsinki and its later amendments.

### Variables (Outcome and exposure)

Microbiological diagnosis SARS-CoV-2 infection was defined by Real-Time Polymerase Chain Reaction of nasal-pharyngeal swab specimen.

The endpoint was in-hospital mortality, assessed either from data at discharge or death certificate. Exposure variables were collected at hospital admission, as follows: (a) demographic and anthropometric characteristics (sex, age, rapid COVID-19 serum test, RT-PCR swab specimen); (b) anamnestic data (number of COVID-19 positives in the family and how many days before hospitalization); (c) symptoms (fever, diarrhea, cough, chest and abdominal pain, anosmia, dysgeusia and related days of onset); previous comorbidities (smoking habit, diabetes, hypertension, chronic cardiac disease, chronic kidney disease (CKD), chronic liver disease (CLD), chronic respiratory disease, neurological disorders or malignancies). Pre-existing comorbidities were evaluated both singularly and according to their presence (absent, 1, 2 and ≥3). In depth, diabetes diagnosis was placed according to the anamnestic records and laboratory exams at admission following American Diabetes Association (ADA) most recent guidelines [11]. A similar approach was used also for hypertension diagnosis [12]. Chronic cardiac diseases, which include ischemic cardiopathy, previous acute myocardial infarction (AMI), heart failure, valvulopathy and atrial fibrillation, were instead diagnosed according to anamnestic data and clinical records. Likewise, also CKD (chronic renal failure) [13], chronic respiratory diseases (chronic obstructive pulmonary disease, asthma, lung fibrosis) [14], neurologic disorders and malignancies. Particularly, as for CLD, chronic hepatopathy from HCV and HBV was diagnosed according to anamnestic records, and laboratory exams at hospitalization. As for cirrhosis, diagnosis was placed considering clinical records and both laboratory and instrumental examination. Finally, NAFLD was diagnosed only based on instrumental and laboratory tests at baseline [15].

All preexisting comorbidities were further weighted calculating the Charlson Comorbidity Index adjusted for age, already tested in COVID-19 patients [16].

At physical examination, data on body temperature, heart and respiratory rates and Acute Respiratory Distress Syndrome (ARDS) Scale (defined as absent, mild, moderate and severe) were also collected. As for Glasgow Coma Score (GCS/15), this was categorized into: Mild impaired consciousness (GCS Score >13), Moderate/Severe impaired consciousness (GCS Score ≤13) and missing data.

In addition, data on laboratory exams at admission were obtained, such as blood cells count, liver, cardiac and kidney function, inflammation indexes and coagulation. As for instrumental exams, instead, either chest computerized tomography (CT), X-Ray or ultrasound were performed to characterize COVID-19 typical lesions.

Also drug therapies, either ongoing or introduced during hospitalization were further collected, as well as respiratory supports throughout the entire period of hospitalization (nasal cannula/Venturi Mask, non-invasive ventilation (NIV) and orotracheal intubation (OTI)) and classified in a Respiratory Severity Scale (RSS).

### Statistical analysis

Categorical data were expressed as number and percentages, whilst continuous variables either as mean and standard deviation (SD) or median and interquartile range (IQR), according to their distribution, previously tested by the Shapiro-Wilk test. The presence of missing data has been managed as reported: a) for continuous data no imputation method, single or multiple, was used—the presence of a missing data has been used as such (NA); b) for categorical data, missing information was treated creating a specific 'missing' category for each variable under analysis. The data were also described considering the number of comorbidities, and also evaluating the statistical association with a p overall to test for any differences among groups of comorbidities (either ANOVA or Kruskal-Wallis test for continuous data, depending on data distribution; Chi-squared or Fisher's exact tests for dichotomous/categorical data, depending on sample size) and p for trend to look for a linear, increasing or decreasing, association trend (nonparametric test for trend across ordered groups or chi-square statistic for the trend). The association between dichotomous variables, in our case the presence or absence of comorbidity, for each possible crossing, was assessed calculating the phi coefficient [17]. Univariable and multivariable logistic regression models were performed to evaluate association between in-hospital mortality and exposure variables. Odds ratios and 95% confidence intervals (OR—95% CI) have been calculated for all models. Variables were included in the multivariable model according the following rules [18]: (a) significance at the univariable analysis (p < 0.05); (b) lack of co-linearity (explored also with preliminary analysis). Comparison between models, to evaluate the improvement and significance of the model, was performed applying the Likelihood-ratio test (LR test), in the case of nested models, or the Bayesian Information Criterion (BIC), in the case of non-nested models, preferring models with lower BIC values. As sensitivity analysis the Firth's correction proposed by van Smeden M et al. was used to increase the accuracy of beta estimates of the model [19]. In addition, the cumulative incidence function (CIF) was calculated, showing the cumulative failure rates over time due to in-hospital mortality. For this particular condition, discharge was considered as a competing event due to the fact that quick recovery and loss to follow-up of patients who are no longer at risk of death from covid-19 should be considered as ‘informative’ censoring. A p-value <0.05 was considered as statistically significant. All analyses were performed using statistical software STATA v16 (StataCorp. 2019. College Station, TX: StataCorp LLC).

## Results

### Characteristics of the study population

662 patients positive at Sars-Cov-2 swab specimen, which required hospitalization, were considered eligible for the study and, of these, 44 were excluded due to incomplete clinical records. 618 patients were finally included in the study, mainly males (61.3%), with a mean age of 65 years (SD 15.2) and a median duration of hospitalization of 20 days [IQR 13–29 days]. The median time elapsed between onset of symptoms and hospitalization was of 4.5 days [IQR 2–7]. At the time of hospitalization, 63.6% of patients did not show any symptom of ARDS, while moderate and severe symptoms were observed, respectively, in about the 13.1% and 7.4%.

In addition, as for the RSS, 330 patients (53.4%) received respiratory support, at the time of hospitalization, either with Venturi mask or nasal cannula. Only the 13% needed either NIV or OTI. 46 patients (7.4%) showed moderate to severe impaired consciousness according to Glasgow Coma Scales (GCS/15). All clinical characteristics at admission are reported in Table 1.

**Table 1:** Baseline characteristics of the study population, both overall and according to the number of preexisting comorbidities.

Parameter	Overall (n = 618)	0 (n = 166)	1 (n = 159)	2 (n = 121)	≥3 (n = 172)	p-overall	p-trend
**Age,** mean (SD)	65 (15.2)	54.8 (14.8)	62.5 (13.8)	69.3 (12.1)	74.0 (12)	<0.001	<0.001
**Sex,** n (%)						0.37	0.123
*M*	379 (61.3)	95 (57.2)	94 (59.1)	80 (66.1)	110 (64)		
*F*	239 (38.7)	71 (42.8)	65 (40.9)	41 (33.9)	62 (36)		
**Any positive in the family,** n (%)	94 (25.4)	36 (30.8)	20 (20.8)	21 (28.4)	17 (20.5)	0.24	0.2
**1**^**st**^ **positive in the family,** n (%)	165 (66.5)	60 (75)	41 (63)	30 (64)	34 (61)	0.27	0.072
**days before hospitalization,** median [IQR]	4.5 [2 – 7]	3 [2 – 7]	5 [2 – 7]	5 [1.5–7]	4 [2 – 6]	0.69	0.75
**Body temp (°C),** mean (SD**)**	37 (0.9)	37.1 (1)	37 (1)	37.2 (1)	36.9 (0.9)	0.12	0.328
**Respiratory rate (apm),** median (IQR)	18 [16 – 22]	18 [14 – 20]	18 [15 – 21]	18 [16 – 22]	18 [16 – 22]	0.066	0.009
**Heart rate (bpm),** mean (SD)	87.0 (15.3)	88.0 (13.7)	89.1 (16.3)	84.7 (13.8)	85.8 (16.6)	0.097	0.012
**Blood pressure (mmHg)**, mean (SD)							
*Systolic*	128.5 (20)	124.9 (16.1)	131.0 (19)	125.7 (19.5)	131.5 (23.5)	0.006	0.129
*Diastolic*	75.2 (11.1)	75.5 (10.1)	76.0 (11.0)	73.6 (9.7)	75.3 (12.9)	0.41	0.499
**Oxygen saturation (%),** median [IQR]	95 [92–97]	96 [93–97]	95 [92–97]	95 [91–97]	94 [91–97]	0.054	0.013
**ARDS Scale**						<0.001	<0.001
*Absent*	393 (63.6)	120 (72.3)	114 (71.7)	73 (60.3)	86 (50)		
*Mild*	98 (15.9)	25 (15.1)	17 (10.7)	17 (14)	39 (22.7)		
*Moderate*	81 (13.1)	17 (10.2)	16 (10.1)	20 (16.5)	28 (16.3)		
*Severe*	46 (7.4)	4 (2.4)	12 (7.5)	11 (9.1)	19 (11)		
**GCS/15, n (%)**						<0.001	<0.001
*Mild impaired consciousness*	448 (72.5%)	129 (77.7%)	118 (74.2%)	87 (71.9%)	114 (66.3%)		
*Moderate/Severe impaired consciousness*	46 (7.5%)	7 (4.2%)	6 (3.8%)	8 (6.6%)	25 (14.5%)		
*Missing*	124 (20.0%)	30 (18.1%)	35 (22.0%)	26 (21.5%)	33 (19.2%)		
**Respiratory Severity Scale,** n (%)						0.007	0.006
*None*	211 (34.1)	73 (44)	58 (36.5)	35 (28.9)	45 (26.2)		
*Mask/Glasses/Cannula*	330 (53.4)	81 (48.8)	74 (46.5)	69 (57)	106 (61.6)		
*NIV*	48 (7.8)	5 (3)	19 (11.9)	11 (9.1)	13 (7.6)		
*OTI*	29 (4.7)	7 (4.2)	8 (5)	6 (5)	8 (4.7)		
**Chronic Cardiac Disease,** n (%)	166 (26.9)	-	18 (11.3)	39 (32.2)	109 (63.4)		
**CKD,** n (%)	67 (10.8)	-	5 (3.1)	8 (6.6)	54 (31.4)		
**Hypertension,** n (%)	302 (48.9)	-	78 (49.1)	82 (67.8)	142 (82.6)		
**Diabetes,** n (%)	113 (18.3)	-	6 (3.8)	25 (20.7)	82 (47.7)		
**Smoking,** n (%)	108 (17.5)	-	18 (11.3)	26 (21.5)	64 (37.2)		
**CLD,** n (%)	35 (5.7)	-	1 (0.6)	10 (8.3)	24 (14)		
**Chronic Respiratory Disease,** n (%)	97 (15.7)	-	16 (10.1)	14 (11.6)	67 (39)		
**Chronic Neurological Disorder,** n (%)	93 (15)	-	8 (5)	24 (19.8)	61 (35.5)		
**Malignancies,** n (%)	53 (8.6)	-	9 (5.7)	14 (11.6)	30 (17.4)		
**CCI Index, median [IQR]**	4 [1–6]	1 [0–2]	3 [1–4]	5 [3–6]	7 [6–10]		
**Laboratory**							
**AST,** median [IQR]	31 [20–46]	33 [22–53.3]	32 [21–49]	33 [20–46]	27 [17.8–42.3]	0.004	0.001
**ALT**, median [IQR]	28 [17–45]	29.5 [20.8–49.8]	30 [19–49]	29 [16.5–44]	21 [13–34]	<0.001	<0.001
**γ-GT,** median [IQR]	38 [22–68]	36 [21–65]	46 [22.5–89]	42 [23–60]	38 [22.8–59]	0.400	0.939
**Total Bilirubin**, median [IQR]	0.6 [0.4–0.87]	0.6 [0.48–0.80]	0.6 [0.4–0.81]	0.68 [0.46–0.97]	0.6 [0.4–0.99]	0.525	0.806

**Abbreviations**: M: Male; F: Female; ARDS: Acute Respiratory Distress Syndrome; GCS: Glasgow Coma Score; RSS: Respiratory Severity Scale; NIV: Non-invasive ventilation; IOT: Orotracheal Intubation; CKD: Chronic Kidney Disease; CLD: Chronic Liver Disease; CCI: Charlson Comorbidity Index; AST: Aspartate Aminotransferase; ALT: Alanine Aminotransferase; γ-GT: Gamma-Glutamyl Transferase; SD: Standard Deviation; IQR: Interquartile Range.

** Chronic cardiac diseases (ischemic cardiopathy, previous acute myocardial infarction (AMI), heart failure, valvulopathy and atrial fibrillation); **CKD** (chronic renal failure, glomerulonephritis and dialysis), **chronic respiratory diseases** (Chronic obstructive pulmonary disease, asthma, lung fibrosis), **CLD** (chronic hepatopathy from HCV and HBV, cirrhosis, NAFLD).

### Pre-existent comorbidities

In our study population almost half of patients had hypertension, whilst cardiovascular diseases (especially atrial fibrillation, acute myocardial infarction and stroke) were reported in the 26.6%. Less prevalent were instead chronic respiratory diseases, CKD and CLD, as well as malignancies, neurologic disorders, smoking habit and diabetes. Overall, 166 patients (27%) were not affected by any comorbidity, whereas 172 (28%) showed either three or more concomitant comorbidities. As shown in Table 1, a stratification by comorbidity number (0, 1, 2, 3 or more) allowed to deepen the description of the clinical features. As well, comorbidities were further weighted by calculating the Charlson Comorbidity Index (CCI) which, as expected, revealed higher in those with either 2 or >3 pre-existing comorbidities on admission.

Very interesting, and partly expected, are the associations (both p-overall and p-trend) of comorbidity number with age, ARDS scale, GCS/15 and RSS.

### In-hospital mortality and clinical prognostic factors

During the observation period, 143 in-hospital mortality events were recorded, with a cumulative incidence of about 23%.

The univariable logistic regression analysis on the association between clinical variables and mortality disclosed a significant association between mortality and age (OR 1.05, 95%CI 1.04–1.07; p<0.001). As well, also Moderate ARDS scale (Moderate vs Absent, OR 2.78, 95%CI 1.52–5.07; p = 0.001) and severe ARDS scale (Severe vs Absent, OR 11.01, 95%CI 5.38–22.53; p<0.001) revealed significantly associated with mortality. As for the respiratory severity scale, also subjects undergone to NIV (NIV vs None, OR 5.81, 95%CI 2.94–11.49; p<0.001) and OTI (OTI vs None, OR 12.90, 95%CI 5.38–30.93; p<0.001) disclosed a significantly higher mortality risk. A moderate-to-severe impaired consciousness at GCS/15 score was associated with a worse clinical outcome (OR 10.12, 95%CI 5.21–19.65; p<0.001).

For what concerns comorbidities already present at admission, several of them were associated with a poor outcome at univariable analysis: Chronic Cardiac Disease (OR 2.05, 95%CI 1.38–3.05; p<0.001), CKD (OR 1.88, 95%CI 1.09–3.23; p = 0.023), CLD (OR 5.67, 95%CI 2.80–11.47; p<0.001), Chronic Respiratory Disease (OR 2.55, 95%CI 1.61–4.04; p<0.001) and Malignant Neoplasm (OR 2.18, 95%CI 1.21–3.93; p<0.010).

Consistently with these findings, a significant association also emerged between a higher mortality rate and the co-presence of at least two comorbidities (OR 2.48, 95%CI 1.35–4.57; p<0.001 for 2 comorbidities and OR 3.70, 95%CI 2.12–6.44; p<0.001 for ≥3). All data are reported in Table 2.

**Table 2:** Univariable Analysis on mortality outcome of baseline characteristics of study population (n = 618).

	Univariable Analysis			
Parameter	OR	95% CI		p
**Age**	1.05	1.04	1.07	<0.001
**Sex**				
* * *F (ref*.*)*	1	-	-	-
* * *M*	1.44	0.97	2.14	0.069
**Body temperature**	1.03	0.83	1.28	0.787
**Respiratory rate**	1.04	1.00	1.08	0.065
**Heart rate**	1.00	0.99	1.02	0.74
**Systolic BP**	0.99	0.98	1.00	0.134
**Diastolic BP**	0.99	0.97	1.01	0.312
**Oxygen saturation**	1.00	0.98	1.01	0.483
**ARDS Scale**				
*Absent (ref*.*)*	1	-	-	-
*Mild*	0.82	0.40	1.67	0.584
*Moderate*	2.78	1.52	5.07	0.001
*Severe*	11.01	5.38	22.53	<0.001
**GCS/15, n (%)**				
*Mild impaired consciousness (ref*.*)*	1	-	-	-
*Moderate/Severe impaired consciousness*	10.12	5.21	19.65	<0.001
*Missing*	2.00	1.26	3.17	0.003
**Respiratory Severity Scale**				
*None (ref*.*)*	1	-	-	-
*Mask/Glasses/Cannula*	1.51	0.95	2.40	0.084
*NIV*	5.81	2.94	11.49	<0.001
*OTI*	12.90	5.38	30.93	<0.001
**Chronic Cardiac Disease**	2.05	1.38	3.05	<0.001
**CKD**	1.88	1.09	3.23	0.023
**Hypertension**	1.30	0.89	1.89	0.175
**Diabetes**	1.48	0.94	2.34	0.092
**Smoking**	1.35	0.84	2.16	0.209
**CLD**	5.67	2.80	11.47	<0.001
**Chronic Respiratory Disease**	2.55	1.61	4.04	<0.001
**Chronic Neurological Disorder**	1.44	0.88	2.36	0.145
**Malignancies**	2.18	1.21	3.93	0.01
**Pre-existing Comorbidities**				
*0*	1	-	-	-
*1*	1.61	0.88	2.94	0.126
*2*	2.48	1.35	4.57	0.004
*>3*	3.70	2.12	6.44	<0.001

**Abbreviations:** M: Male; F: Female; BP: Blood Pressure; ARDS: Acute Respiratory Distress Syndrome; GCS: Glasgow Coma Score; RSS: Respiratory Severity Scale; CKD: Chronic Kidney Disease; CLD: Chronic Liver Disease; NIV: Non-invasive ventilation; IOT: Orotracheal Intubation; OR: Odds Ratio; CI: Confidence Interval.

The subsequent multivariable model was based on the following concepts and rules: 1) the variables statistically significant at the univariable model were included; 2) we decided to consider and correct also for sex; 3) as we aimed to evaluate the single comorbidities, we did not include the variable ‘comorbidity number’, after appropriate evaluation, in the preliminary analysis with the statistics phi, that none of the single comorbidities showed high levels of correlation; 4) in the choice between ARDS scale and RSS, given the overlap of clinical nature, we chose the best univariable model evaluated with the BIC score.

The significance emerged at univariable analysis was partially confirmed at the multivariable, with the addition of sex (Male vs Female -ref-; OR 2.63, 95%CI 1.42–4.90; p = 0.001). Of note, CLD (OR 5.88, 95%CI 2.39–14.46; p<0.001) and malignancies (OR 2.62, 95%CI 1.21–5.68; p = 0.015) disclosed an independent association with a poor prognosis. Likewise, also NIV/OTI respiratory supports revealed independent associated with a higher in-hospital mortality (OR 4.63, 95%CI 1.89–11.34; p<0.001 and OR 6.65; 95%CI 1.93–22.99; p = 0.003, respectively), as well as a moderate-to-severe impaired consciousness at GCS/15 (OR 7.26, 95%CI 3.43–15.37; p<0.001).

A sensitivity analysis, excluding comorbidities not statistically significant in the previous model, further confirmed the observed associations. Both multivariable models are shown in Table 3.

**Table 3:** Multivariable models on mortality outcome in our COVID-19 study cohort (n = 618).

	Multivariable				Multivariable—Sensitivity			
Parameter	OR	95% CI		p	OR	95% CI		p
**Age**	1.05	1.02	1.07	<0.001	1.05	1.03	1.07	<0.001
**Sex**								
*F (ref*.*)*	1	-	-	-	1	-	-	-
*M*	2.63	1.42	4.90	0.002	2.70	1.46	4.98	0.001
**GCS/15, n (%)**								
*Mild impaired consciousness (ref*.*)*	1	-	-	-	1	-	-	-
*Moderate/Severe impaired consciousness*	7.26	3.43	15.37	<0.001	7.56	3.60	15.89	<0.001
*Missing*	1.91	1.10	3.31	0.021	1.92	1.11	3.32	0.02
**Respiratory Severity Scale**								
*None (ref*.*)*	1	-	-	-	1	-	-	-
*Mask/Glasses/Cannula*	0.97	0.51	1.83	0.919	0.98	0.52	1.84	0.943
*NIV*	4.63	1.89	11.34	0.001	4.72	1.94	11.50	0.001
*OTI*	6.65	1.93	22.99	0.003	6.82	1.99	23.45	0.002
**Chronic Cardiac Disease**	0.96	0.53	1.76	0.905				
**CKD**	1.01	0.44	2.34	0.974				
**CLD**	5.88	2.39	14.46	<0.001	5.87	2.41	14.29	<0.001
**Chronic Respiratory Disease**	1.22	0.61	2.44	0.569				
**Malignancies**	2.62	1.21	5.68	0.015	2.58	1.19	5.57	0.016

**Abbreviations:** M: Male; F: Female; ARDS: Acute Respiratory Distress Syndrome; GCS: Glasgow Coma Score; RSS: Respiratory Severity Scale; CKD: Chronic Kidney Disease; CLD: Chronic Liver Disease; NIV: Non-invasive ventilation; IOT: Orotracheal Intubation; OR: Odds Ratio; CI: Confidence Interval.

As an additional sensitivity analysis the Firth's correction was used, this analysis showed similar results of the reported models.

**Fig 2:**
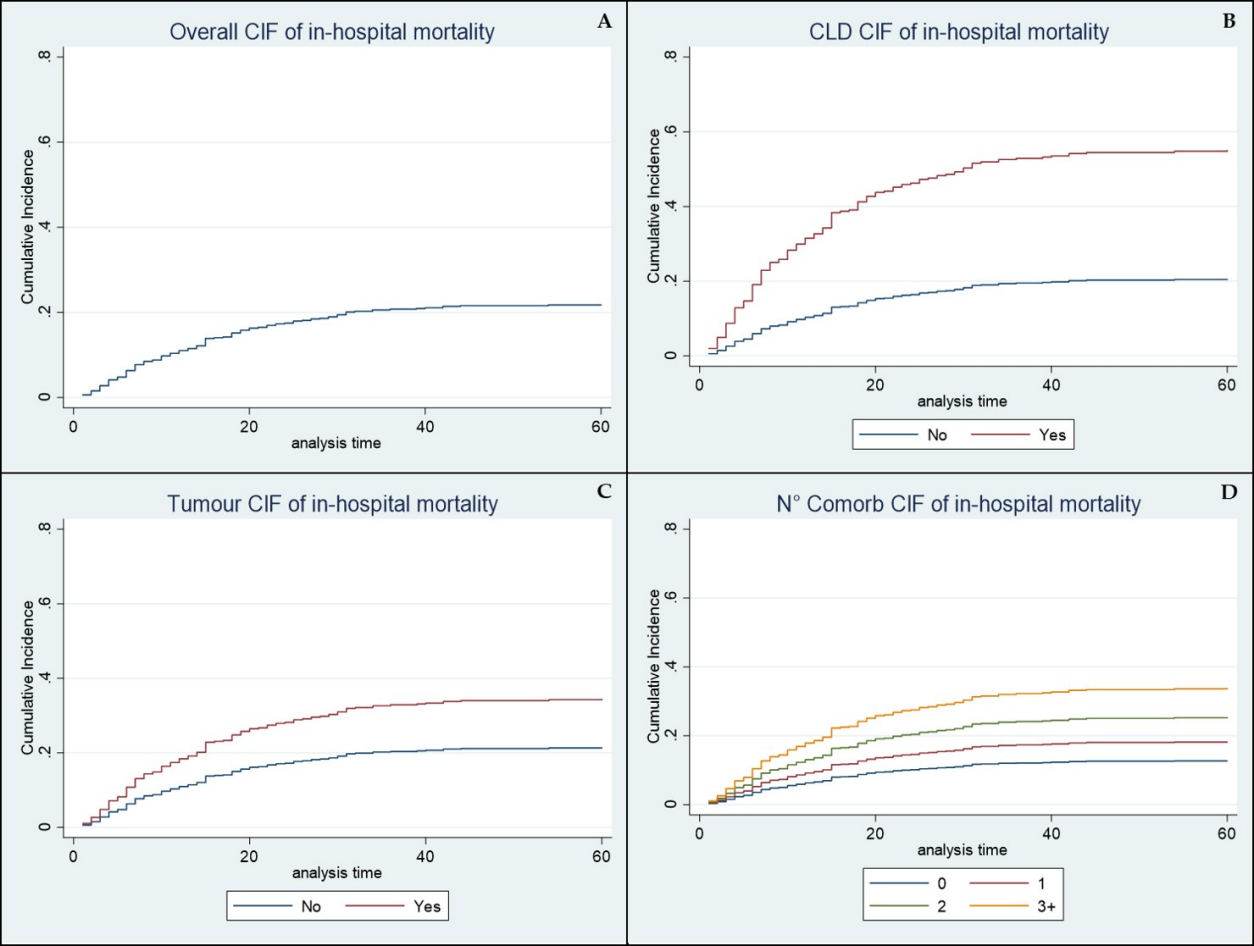
Cumulative incidence functions (CIFs) of overall comorbidities (Panel A), Chronic Liver Disease (Panel B), malignancies (Panel C) and number of coexisting comorbidities (Panel D).

Cumulative incidence of the study population, both overall and subdivided according to the number of coexisting comorbidities and of those independently associated with a worse prognosis are further described in Fig 2 (Panels A→D).

The significant association between CLD and poor COVID-19 prognosis, confirmed both at multivariable and sensitivity analysis, have prompted us to focus on the subgroup with CLD, mainly characterized by either viral or metabolic hepatopathies. In particular, of these, the 45.7% were affected by Chronic C Hepatitis, 37.1% had Non-alcoholic Fatty Liver Disease (NAFLD), whilst the remaining 17.2% had Chronic B Hepatitis. None of them had hepatocellular carcinoma, acute liver failure, autoimmune liver diseases or other forms of CLD. The major part has a Child–Pugh score A (51.4%) or B (42.9%) score, whilst only two disclosed a Child–Pugh score C (5.7%). All clinical characteristics at baseline are shown in S1 Table.

We further consider modifications of liver function indexes in this subgroup according to the clinical outcome. Both Aspartate and Alanine aminotransferase (AST and ALT), γ-glutamyl transferase (γ-GT) and bilirubin were almost similar at baseline between alive and dead patients, whilst at end of hospitalization, there was a significant increase of all these indexes among dead patients; in particular of total bilirubin (p = 0.0011), AST (p = 0.020), ALT (p = 0.016), and γ-GT (p = 0.002). Whole data are shown in S1 Table.

Consistent with these findings, a significant association between worsening of liver function and mortality was further observed in the entire study population (S2 Table).

## Discussion

The still current absence of specific therapies approved by National and International Health Regulatory Authorities, 8 months after the WHO declaration of a pandemic, requires us to identify which clinical conditions affect the outcome of patients. This aspect is mandatory to identify subjects at greatest risk, for whom hospitalization must be early and management very close.

COVOCA, to date the largest multicenter study in terms of sample size in the Campania region involving COVID-19 hospitalized patients, presents several insights.

The association between different comorbidities and mortality in COVID-19 patients has already been partially investigated [20–24].

In particular, in our study cohort chronic comorbidities disclosed a prevalence almost comparable to that from previous evidence [25]. In depth, hypertension was the most prevalent (48.9%), followed by chronic cardiac disease (26.9%), diabetes (18.3%), chronic respiratory disease (15.7%), chronic neurological disorders (15%), CKD (10.8%), malignancies (8.6%) and CLD (5.7%). In acute we considered GCS/15 and RSS.

In our study we observed an independent association between in-hospital mortality and poor clinical conditions on admission. In particular, RSS (in patients either in NIV or OTI, OR 4.63 and 6.65 respectively), CLD (OR 5.88) and malignancies (OR 2.62) revealed strong predictor of mortality. As well, a moderate-to-severe impaired consciousness at GCS/15 revealed a negative prognostic element, thus confirming the reliability of this parameter also in COVID-19, as suggested by other authors [25].

This finding is consistent with previous studies focused on the clinical effects of NIV/OTI on patients’ prognosis [26,27]. However, this topic still remains controversial and thus it deserves further insights [28,29].

Of note, an association emerged between mortality and number of comorbidities, with a 3.7 times higher risk of mortality among those who had at least 3 comorbidities vs those who had none, suggesting that COVID-19 patients with several comorbidities are more frail. This finding seems consistent with data from large studies conducted in Northern Italy [30], as well as with national reports of “Istituto Superiore di Sanità (ISS)” [31]. This finding may be explained by the dysregulation of main physiologic systems (pituitary-hypothalamus-adrenal, immune and sympathetic systems) triggered by the presence of multiple comorbidities. Such hypothesis has been recently proposed as a cause of the increase mortality rate in COVID-19 patients with multiple comorbidities [21,32].

Surprisingly, in our study the significant association of both CKD and cardiovascular diseases came lost on multivariable analysis. Such finding might have been affected by a not excessively low GFR (mean 35.8 ml/min/1.73m^2^) as well as by the lack of information on the ventricular ejection fraction does not allow us to establish whether it was conserved or not.

The CLD incidence observed in our study population seems comparable to that described in previous reports [33–36]. Worthy of note is the association between mortality and CLD (OR 5.88), finding strongly enhancing data reported in a recent US large observational study which registered almost a 3-times increased mortality risk [37].

Actually, the relationship between CLD and COVID-19 outcome has been investigated in very few studies, from which an association between a worse outcome and chronic hepatopathy emerged [2,10,38].

We observed almost in all CLD patients survived at the end of hospitalization a Child–Pugh score A, while the deceased had either a Child–Pugh score B or C. Moreover, CLD patients died earlier than the overall COVID population, confirming that chronic liver disease rapidly worsens the prognosis of this patients’ setting.

Originally, we observed during hospitalization an increase of hepatic damage (assessed by AST, GGT and bilirubin) both in CLD subjects with poor prognosis, as well as in overall COVID dead patients too.

Currently, the reason for a worse prognosis of COVID-19 patients with a pre-existing hepatopathy still remains unclear, as well as the role of COVID-19 in the exacerbation of liver disease. Based on our findings, we could hypothesize that patients with CLD at an advanced stage are already characterized by a worse clinical condition, which negatively affects their prognosis. However, also a dysregulation of the innate immune response may represent an aspect of liver injury in COVID-19, which would enhance the risk of mortality in this subsetting [39].

As for the innate immune response, several pathophysiologic mechanisms have been assumed, such as the direct cytotoxic effect of the virus on the liver, mediated by the angiotensin converting enzyme 2 (ACE2) expressed in the cytomembrane [40]. It has also been observed that an interaction of the virus with Kupffer cells and intra-hepatic T lymphocytes could stimulate a liver damage [41,42]. A further hypothesis concerns the systemic reaction induced by the virus, which would activate inflammatory molecules such as IL-6 and TNF-alpha, thus triggering a liver damage [34,43–45].

An interesting new hypothesis could involve an imbalance between free radicals and the main antioxidants, in particular the hepatic glutathione (GSH), decreased in chronic liver disease [46].

Due to the observation that several viral infections are characterized by a decrease in GSH, many *in vitro* studies have been performed, showing that GSH can attack viruses with different replicative mechanisms (HIV and other retroviruses, influenza and parainfluenza viruses, rhinovirus and HSV-1) and inhibit viral replication at different stages [47,48].

Intriguingly, on these bases, low levels of GSH have been recently suggested among the major causes of the excessive inflammatory response linked to severe COVID-19 outcome [48]. This could represent a possible rationale for high mortality in COVID-19 patients with CLD.

Moreover, some studies showed that most of drugs used in COVID-19 treatment, especially antivirals, could either determine a liver damage or re-activate latent viral diseases [49].

This study presents several limitations. Firstly, beyond its retrospective observational design, is the low number of CLD patients. Though the remarkable statistical significance and the support from the previous evidence, the limited sample size of CLD subpopulation did not allow to further assess which liver disease was majorly associated with mortality. As well, we could not assess data on BMI as most of Centers could not supply this information due to clinical conditions of patients. These missing data did not allow the inclusion of BMI among risk factors as well as its impact on a poor outcome [24]. As well, it would been useful to consider previous socio-economic status, as reported in diverse papers [24].

On the other hand, the methodological rigor of the analysis allows us to deem our findings as reliable and generalizable even on larger populations.

## Conclusion

Outcome of patients hospitalized for COVID-19 appears strongly affected by both clinical conditions on admission (GCS and RSS) and comorbidities. Notably, their association may reduce patients' ability to overcome the infection and the complications it triggers. Pre-existing chronic liver disease, especially in the moderate-advanced phase, appears relevant in significantly worsening prognosis of hospitalized patients. Actually, this could be due to key role of liver in the biosynthesis of molecules involved in coagulation balance and inflammatory response.

Further research is mandatory to deepen and expand the study of prognostic factors in hospitalized patients [50] and to evaluate at medium/long term the effect of COVID-19 on the progression of comorbidities, in particular CLD, in patients who have overcome the infection.

## Supporting information

S1 Table Characteristics of CLD subpopulation at Baseline and at discharge/death (n = 35). (DOCX)

S2 Table Baseline and discharge liver function indexes modifications in the entire study population (n = 618). (DOCX)
