# Supplementary material for: Impact of chronic liver disease upon admission on COVID-19 in-hospital mortality: Findings from COVOCA study
Source: PLoS One. 2020 Dec 10;15(12):e0243700. doi: 10.1371/journal.pone.0243700 (PMC7728173; doi:10.1371/journal.pone.0243700)
Supplement: S1 Table — (DOCX) [file pone.0243700.s001.docx]

| **Supplementary Table 1.** Characteristics of CLD subpopulation at Baseline and at Discharge/Death (n=35). | | | | |
| --- | --- | --- | --- | --- |
| **Parameter** | **Overall** | **Alive (n=14)** | **Dead (n=21)** | **p** |
| ***Baseline*** |  |  |  |  |
| Age | 72 [59-81] | 69.5 [62.5 – 78] | 74 [58.5 – 83.5] | 0.583 |
| Child–Pugh score, n (%)  *A*  *B*  *C* | 18 (51.4)  15 (42.9)  2 (5.7) | 10 (71.4)  4 (28.6)  0 (-) | 8 (38.1)  11 (52.4)  2 (9.5) | 0.119 |
| AST, median [IQR] | 35 [23-47] | 35 [23.75 – 51.25] | 37 [21.5 – 48] | 1.000 |
| ALT, median [IQR] | 25 [15-37] | 25.5 [17 – 41.5] | 23 [15 – 35] | 0.561 |
| γ-GT, median [IQR] | 46 [34-72] | 44.5 [34.25 – 58.85] | 46 [33.5 – 92.5] | 0.678 |
| Total bilirubin, median [IQR] | 2.1 [1.20-4.50] | 1.06 [0.58 – 2.17] | 1.30 [0.68 – 1.88] | 0.583 |
| Duration of hospitalization, median [IQR] | 19 [10.8-26.5] | 24 [19.8 – 28] | 15 [8 – 19.8] | 0.004 |
| ***Discharge/Death*** |  |  |  |  |
| AST, median [IQR] | 45 [23 – 65] | 34.5 [20 – 48.25] | 50 [39 – 108.5] | 0.020 |
| ALT, median [IQR] | 30 [18 – 62] | 22 [14.75 – 28.5] | 50 [23 – 95.5] | 0.016 |
| γ-GT, median [IQR] | 88 [42 – 311] | 42.5 [32.75 – 75.25] | 162 [83.5 – 323] | 0.002 |
| Total Bilirubin, median [IQR] | 2.10 [1.20 – 4.5] | 1.61 [0.90 – 2.77] | 2.9 [1.83 – 5.7] | 0.011 |
| **Abbreviations**: AST: Aspartate aminotransferase; ALT: Alanine aminotransferase; γ-GT: γ-glutamyl transferase; IQR: Interquartile Range | | | | |
