# Supplementary material for: Impact of chronic liver disease upon admission on COVID-19 in-hospital mortality: Findings from COVOCA study
Source: PLoS One. 2020 Dec 10;15(12):e0243700. doi: 10.1371/journal.pone.0243700 (PMC7728173; doi:10.1371/journal.pone.0243700)
Supplement: S2 Table — (DOCX) [file pone.0243700.s002.docx]

| **Supplementary Table 2.** Baseline and Discharge liver function indexes modifications in the entire study population (n=618). | | | |
| --- | --- | --- | --- |
| **Parameter** | **Alive**  **(n=475)** | **Dead**  **(n=143)** | **p** |
| **Baseline** |  |  |  |
| **AST,** median [IQR] | 30 [20 – 45] | 34 [21 – 50.75] | 0.042 |
| **ALT**, median [IQR] | 28 [17 – 45] | 28.5 [16 – 44.25] | 0.897 |
| **γ-GT,** median [IQR] | 37 [20 – 68.5] | 46 [31 – 69.5] | 0.048 |
| **Total Bilirubin**, median [IQR] | 0.60 [0.40 – 0.80] | 0.73 [0.44 – 1.20] | 0.001 |
| **Discharge/Death** |  |  |  |
| **AST,** median [IQR] | 23 [16 – 36] | 44 [23 – 75] | <0.001 |
| **ALT**, median [IQR] | 26.5 [16 – 48.75] | 41 [25 – 62] | <0.001 |
| **γ-GT,** median [IQR] | 36 [20 – 58] | 93 [42.5 – 192.5] | <0.001 |
| **Total Bilirubin**, median [IQR] | 0.53 [0.40 – 0.79] | 1.20 [0.70 – 2.90] | <0.001 |
| **Abbreviations**: AST: Aspartate aminotransferase; ALT: Alanine aminotransferase; γ-GT: γ-glutamyl transferase; IQR: Interquartile Range | | | |
